# Progressive Metabolic Abnormalities Associated with the Development of Neonatal Bronchopulmonary Dysplasia

**DOI:** 10.3390/nu14173547

**Published:** 2022-08-28

**Authors:** Chengyin Ye, Jinghua Wu, Jonathan D. Reiss, Tiffany J. Sinclair, David K. Stevenson, Gary M. Shaw, Donald H. Chace, Reese H. Clark, Lawrence S. Prince, Xuefeng Bruce Ling, Karl G. Sylvester

**Affiliations:** 1 Department of Health Management, School of Public Health, Hangzhou Normal University, Hangzhou 311100, China; 2 Department of Pediatrics, Stanford University School of Medicine, Stanford, CA 94304, USA; 3 Stanford Metabolic Health Center, Stanford Children’s Hospital, Stanford, CA 94304, USA; 4 Department of Surgery, Division of Pediatric Surgery, Stanford University School of Medicine, Stanford, CA 94304, USA; 5 Medolac Laboratories, Boulder City, NV 89005, USA; 6 Pediatrix-Obstetrix Center for Research, Education and Quality, Sunrise, FL 33323, USA; 7 Clinical and Translational Research Program, Betty Irene Moore Children’s Heart Center, Lucile Packard Children’s Hospital, Palo Alto, CA 94304, USA

**Keywords:** neonatal bronchopulmonary dysplasia, prematurity, newborn metabolic profiling, amino acids, acylcarnitine

## Abstract

**Objective:** To assess the longitudinal metabolic patterns during the evolution of bronchopulmonary dysplasia (BPD) development. **Methods:** A case-control dataset of preterm infants (<32-week gestation) was obtained from a multicenter database, including 355 BPD cases and 395 controls. A total of 72 amino acid (AA) and acylcarnitine (AC) variables, along with infants’ calorie intake and growth outcomes, were measured on day of life 1, 7, 28, and 42. Logistic regression, clustering methods, and random forest statistical modeling were utilized to identify metabolic variables significantly associated with BPD development and to investigate their longitudinal patterns that are associated with BPD development. **Results:** A panel of 27 metabolic variables were observed to be longitudinally associated with BPD development. The involved metabolites increased from 1 predominant different AC by day 7 to 19 associated AA and AC compounds by day 28 and 16 metabolic features by day 42. Citrulline, alanine, glutamate, tyrosine, propionylcarnitine, free carnitine, acetylcarnitine, hydroxybutyrylcarnitine, and most median-chain ACs (C_5_:C_10_) were the most associated metabolites down-regulated in BPD babies over the early days of life, whereas phenylalanine, methionine, and hydroxypalmitoylcarnitine were observed to be up-regulated in BPD babies. Most calorie intake and growth outcomes revealed similar longitudinal patterns between BPD cases and controls over the first 6 weeks of life, after gestational adjustment. When combining with birth weight, the derived metabolic-based discriminative model observed some differences between those with and without BPD development, with c-statistics of 0.869 and 0.841 at day 7 and 28 of life on the test data. **Conclusions:** The metabolic panel we describe identified some metabolic differences in the blood associated with BPD pathogenesis. Further work is needed to determine whether these compounds could facilitate the monitoring and/or investigation of early-life metabolic status in the lung and other tissues for the prevention and management of BPD.

## 1. Introduction

Bronchopulmonary dysplasia (BPD), the most common acquired morbidity of preterm infants, is a severe form of chronic lung disease and is generally characterized by the presence of chronic respiratory insufficiency and a supplemental oxygen requirement at 28 days of life with consistent radiologic changes or continuing need for oxygen therapy at 36 weeks’ postmenstrual age [1]. The incident rate of BPD can reach 30% for extremely premature infants born before 30 weeks of gestational age (GA) [2,3]. Infants with BPD typically require advanced respiratory support including mechanical ventilation, oxygen therapy, and numerous medications during initial hospitalization and beyond. Infants with BPD are at increased risk for hospital readmission and lethality from additional respiratory illnesses exacerbating poor lung function in early life. The long-term complications of BPD can also be severe and can include compromised pulmonary function, pulmonary hypertension, asthma-like symptoms, and exercise intolerance with altered response to hypoxia [4]. Taken together, the overall morbidity associated with BPD is a persistent and a substantial burden for the individual and the healthcare system [5].

In recent studies, metabolic signatures of acylcarnitines (ACs) and amino acids (AAs) have been identified in newborn screen metabolic panels in association with metabolic disorders and critical illnesses in mature/premature infants, including fatty acid oxidation disorders, carnitine cycle disorders, cardiac malformation/malfunction, gastrointestinal disorders, and respiratory distress [6,7,8,9,10,11]. Evidence has suggested that fatty acid metabolism may be associated with processes of lung injury and repair, inflammation, and immune modulation and therefore could be utilized as biomarkers for screening BPD and other acquired diseases of the preterm newborn [12]. Several amino acid profiles, such as decreased citrulline, arginine, and tyrosine levels and elevated phenylalanine levels at birth, have previously been found to be significantly associated with an increased risk of BPD and its complications, such as pulmonary hypertension [13,14,15,16,17]. Prior observations obtained beyond the newborn period in children have also found a relationship between carnitine metabolism and pulmonary vascular disease [18].

To identify disruptions in metabolism involving protein and fatty acid metabolism that are linked to acute or chronic disorders, our group has previously identified associations between either at-birth levels or the serial progression of specific ACs and AAs and the development of necrotizing enterocolitis (NEC) in preterm newborns [11,19]. Following the same rationale, we hypothesized that longitudinal changes in metabolic intermediates (i.e., ACs and AAs) could be utilized to identify metabolic differences associated with the development of BPD in preterm newborns and may potentially facilitate the investigation of early-life metabolic disparities in the lungs and other tissues between BPD cases and preterm babies without BPD.

## 2. Methods

### 2.1. Study Cohort

A total of 995 preterm infants were derived from a prospective clinical study that evaluated metabolic profiles in premature infants. Twenty-three sites in 17 states managed by the Pediatrix Medical Group enrolled patients in this study between April 2009 and September 2012, and the original protocol was approved by both the Western Institutional Board and/or each site’s local Institutional Board [11,20]. The GA of these infants ranged from 23 to 31 weeks and was based on obstetrical estimates and consistent with developmental physical examination by a neonatologist. After birth, infants’ clinical data were collected, and their longitudinal nutrition-related variables and metabolic samples were continuously followed over the first six weeks of life, at days 1 (within 24 h of delivery), 7 (7–8), 28 (27–29), and 42 (41–43). The methods for data collection were previously described [11]. At each time point, the dried blood spots of infants were obtained and assayed for a panel of 72 metabolites using tandem mass spectrometry for newborn screening analysis. The metabolic panel consisted of 14 AAs, 9 AA ratios, 35 ACs, 12 AC ratios, and 2 combined AA + AC measures. The metabolic testing was performed at a central laboratory (PerkinElmer Genetics, Bridgeville, PA) as previously described [11,19]. Demographic data was collected using the electronic data capture system, and clinical research associates monitored each site for adherence to the protocol and data accuracy.

In this study, the infants were all born <32 weeks and the diagnosis of BPD was defined as meeting BPD criteria of oxygen dependency at 28 days of life. A total of 355 infants were recognized as candidates of cases. Control newborns were selected if they meet the following criteria: (1) no diagnosis of any type of intraventricular hemorrhage (IVH), retinopathy of prematurity (ROP), NEC, or BPD during the first six weeks of life; and (2) survival at the end of the study (day 42 of life). Specifically, IVH was diagnosed if an infant had any of subependymal (grade 1), intraventricular (grade 2), intraventricular with dilation (grade 3) or intraparenchymal (grade 4), while ROP was determined as either ROP 1, 2, 3, or ROP surgery. NEC was indicated if an infant had a NEC-medical or NEC-surgical record. A NEC-medical record was present if an infant had at least one of the following clinical signs: bilious gastric aspirate or emesis, abdominal distention, or blood in stool without evidence of a rectal fissure and had one or more of the following radiographic findings: pneumatosis intestinalis, hepatobiliary gas, or pneumoperitoneum, while a NEC-surgical record indicated that a surgery was carried out following the NEC diagnosis [11]. As a result, 395 infants were recognized as candidates of controls. After reviewing the GA distribution among cases and controls, it was found that most cases (56.90%) were born at a lower GA (ranged from 23 to 26 weeks) than most controls (with 68.86% delivered at GA of 29–30 weeks). The number of cases and controls were only balanced in 27–28-week gestation, thus, in order to eliminate possible poor growth effects attributed to a lower GA in cases, we formed the sub-cohort and only recruited 97 cases and 108 controls who were born at a matched GA of 27–28 weeks for the following metabolic analysis of BPD development, along with their 758 data points collected over the first six weeks of life (Figure S1).

### 2.2. Statistical Analysis

All statistical analysis was conducted using R software. Demographic and clinical characteristics were statistically compared between BPD newborns and controls using a *t*-test for continuous variables and a chi-square test or Fisher’s exact test for categorical variables. In the first 42 days of life, loess curves were drawn for each nutrition-related variable in the BPD and control sub-cohorts, respectively, to reveal different longitudinal patterns. Among these nutrition-related measurements, the infant’s weight-adjusted daily calorie intake was calculated as calories received per kg per day from all nutrition on a certain time interval (i.e., day 1, 7, 28, and 42 after delivery), denoted as calories (cal/kg/day). Similarly, the infant’s weight (g) variable was assessed on each indicated day of measurement.

Logistic regressions were applied at each time point to capture the subset of metabolic variables significantly associated with BPD development, using the GA-matched sub-cohort, and the global FDR was used to adjust for multiple comparison testing. Next, fold-changes between cases and controls were further calculated over time for those identified significant metabolites, and a hierarchical clustering heatmap was generated accordingly using R pheatmap and corrplot packages. Lastly, we investigated whether the captured metabolic panel could be used to describe metabolic disparities between infants with and without BPD over the first several weeks of life. The R randomForest package was used for modeling where most parameters were set by default and the number of trees was optimized as 400. Herein, we used the entire dataset of 355 BPD cases and 395 control infants regardless of their GA and randomly and evenly separated the dataset into construction and validation cohorts with a ratio of 2:1 and further constructed the discriminative models using random forest, by considering the subset of significant metabolites, as well as other leading characteristics at birth (i.e., birth weight, GA, and sex). These discriminative models’ performance was evaluated and compared using c-statistics.

## 3. Results

After excluding preterm infants who had been diagnosed with NEC, ROP, or IVH, a total of 355 BPD cases and 395 control infants were enrolled in the primary cohort, whose characteristics are summarized in Table S1. In this primary cohort, infants who developed BPD were more premature than controls, resulting in significantly lower birth weights and earlier GA. Therefore, to identify metabolic indicators associated with BPD development while eliminating growth faltering attributed to a lower GA in the following study, we matched infants’ GA and formed a new sub-cohort that consisted of 97 BPD cases and 108 control infants who were born at GA of 27–28 weeks. In this sub-cohort, the cases and controls showed no significant difference in sex, race, mode of delivery, multiple-gestation counts, and being small for gestational age (SGA) (Table 1). However, even with the matched GA, infants who developed BPD still had lower birth weights and a higher rate of patent ductus arteriosus (PDA).

**Figure 1:**
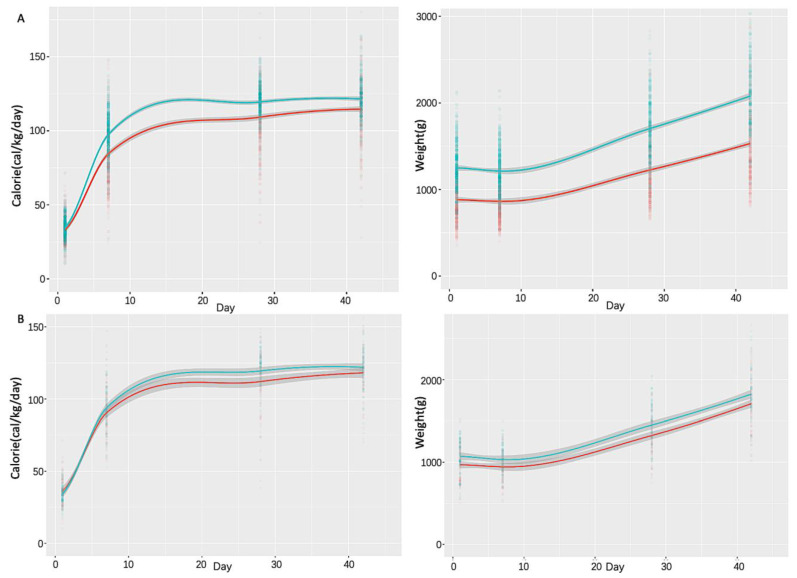
Longitudinal comparisons of body weight and weight-adjusted calories received over the first 6 weeks of life between BPD cases and control infants of (**A**) the primary cohort and (**B**) the sub-cohort whose gestational age (GA) ranged from 27 to 28 weeks.

The longitudinal patterns of body weight and weight-adjusted calories received were measured on day of life 1, 7, 28, and 42 and compared between newborns who developed BPD and controls for both the primary cohort without GA matching (Figure 1A) and the 27–28-week GA sub-cohort (Figure 1B). The results from the entire cohort revealed that newborns developing BPD consistently received fewer overall weight-adjusted total calories than controls during the first 42 days of life and that newborns that developed BPD were smaller than controls in terms of body weight. However, after matching GAs in the 27–28-week sub-cohort, only small statistical differences were observed between BPD cases and control infants for the overall weight-adjusted total calories (on day 28 of life) and body weight (on day 28 and 42 of life).

In order to identify metabolic indicators associated with BPD development, while controlling the confounders related to malnutrition and growth faltering, a focal analysis on the 27–28-week GA sub-cohort was pursued using logistic regression by treating birth weight as a covariate to identify metabolic variables that were associated with BPD development at at least one time point during the period of analysis. The global FDR of 0.05 was adopted for multiple comparison adjustment. As a result, among the 72 longitudinally measured metabolic variables, an intersection of 27 metabolic analytes were captured, which led to a compressed metabolic panel representing the difference between infants that developed BPD and controls over the first 42 days of life. Since both birth weight and GA were carefully controlled, the recognized metabolic panel was independent from confounders of malnutrition and poor growth and may be taken as BPD associators independent of established risk factors. During the first day of life, no significant metabolic analytes were noted to be different between cases and controls. Subsequently, the significant metabolites gradually increased from 1 predominant AC metabolic differentials by day 7 to 19 significant AA and AC differentials by day 28 and reduced to 16 metabolic features by day 42 (Table S2 and Figure S2).

**Figure 2:**
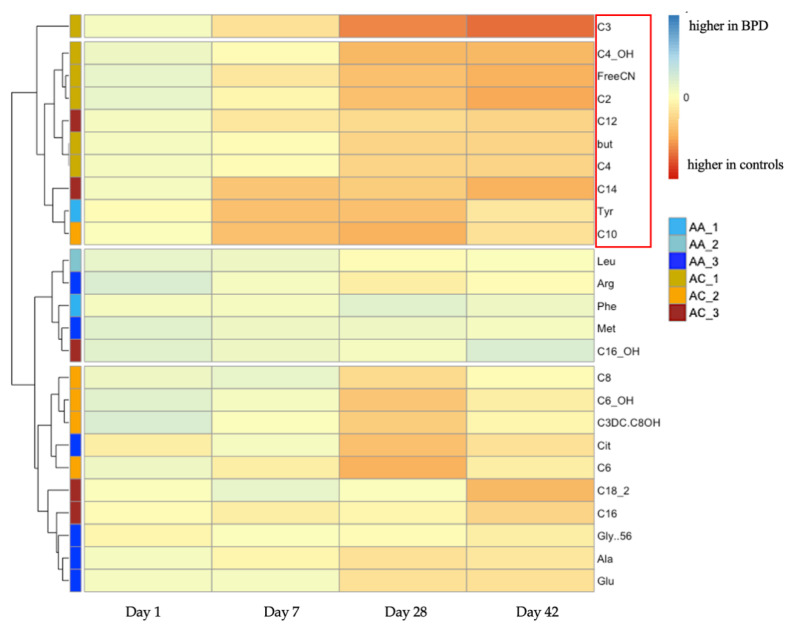
Hierarchical clustering heatmap of 25 distinct metabolites depicting their fold change in the ratio between newborns with BPD and without BPD (log_2_FC) at each time point. Those metabolites in the red frame were the most significantly higher in BPD cases. FC: fold change.

Since the identified metabolic panel contains several combined variables from different metabolites, we further extracted and generated a subset of 25 distinct metabolites (Table S3) involved in the 27 significant variables. These 25 metabolites were further investigated to reveal their longitudinal patterns and capture these up- or down-regulated metabolites in infants with BPD, providing evidence for underlying metabolic pathways that may be associated with BPD development. Firstly, at each time point, the fold change of each metabolite between newborns developing BPD and controls was calculated and aggregated by hierarchical clustering (Figure 2). The clusters were primarily split into four categories of down-regulated and up-regulated clusters. In terms of ACs, propionylcarnitine (C3) is the most significantly down-regulated AC in BPD cases, forming an exclusive cluster. Additionally, most short-chain ACs (i.e., free carnitine (FreeCN), acetylcarnitine (C2), and hydroxybutyrylcarnitine (C4_OH)) and median-chain ACs (C5:C10) displayed a longitudinally down-regulated pattern in BPD cases since day 7 of life. For long-chain ACs, there was a variable pattern of both decreased and increased levels observed in BPD babies. For instance, the long-chain AC of hydroxypalmitoylcarnitine (C16_OH) was up-regulated significantly at day 1 and 42 of life, yet others were down-regulated in BPD infants. In terms of amino acids, we found citrulline (Cit), alanine (Ala), and glutamate (Glu) were longitudinally down-regulated in cases since day 28 or day 7 of life. Tyrosine (Tyr) was dramatically decreased since day 7 of life in BPD infants, whereas phenylalanine (Phe) and methionine (Met) were slightly increased in BPD infants over the first six weeks. Secondly, to further explore this metabolic disparity, we calculated the pairwise correlation over the four time points for each two of the identified metabolites within the case and within the control groups (Figure S3). It was found that, for ACs, C12 had a similar pattern to C16 and C14 in controls but aggregated with C6_OH in cases. C8 and C18_2 formed one cluster in cases but went to two distinct clusters in controls. Meanwhile, for amino acids, Cit and Tyr aggregated into one cluster in the controls but belonged to two different clusters in the BPD cases, as Tyr aggregated with Glu, Ala, and Gly to form a new cluster in cases.

**Figure 3:**
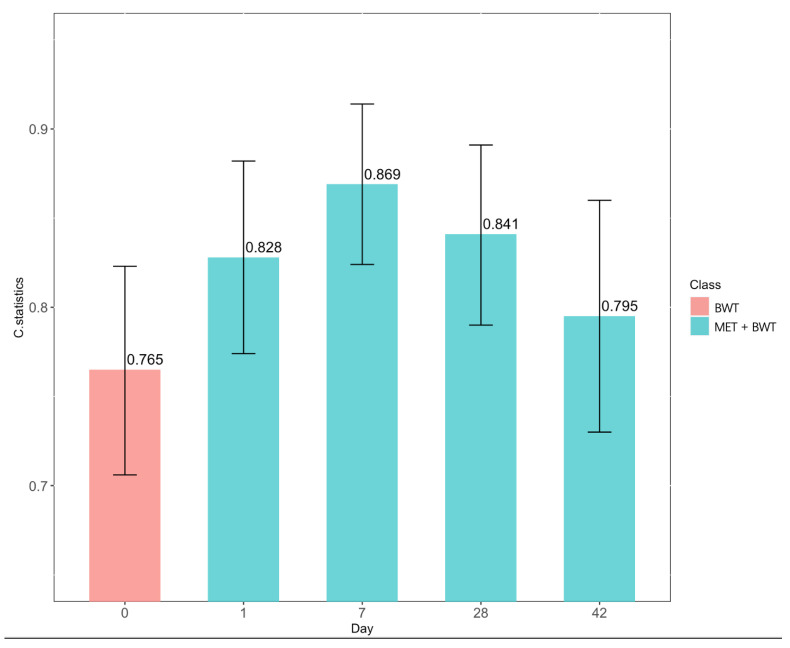
The c-statistics and its 95% CI of the built discriminative models on the 1/3 validation cohort at each time point, utilizing the observed metabolic panel as predictors. MET: the observed metabolic panel. BWT: birth weight.

Finally, we treated this identified metabolic panel of 27 analytes from day 1 to day 42 of life as input and built discriminative models together with birth weight to examine whether the panel could be used to describe certain metabolic differences between infants with and without BPD, facilitating the monitoring of BPD development in early life. To increase the sample size and modeling accuracy, the primary cohort consisting of 355 BPD cases and 395 control infants were ultilized at this modeling stage. After randomly splitting the dataset into the development and validation subsets with a ratio of 2:1, we built the models using random forest algorithms. Results (Figure 3) showed that, on the validation subset, when combining with birth weight, the metabolic panel attained a modest classification accuracy at birth (c-statistic = 0.828), which immediately increased to a better discriminative ability (c-statistic = 0.869) on day 7 of life and day 28 of life (c-statistics = 0.841) and then dropped to a reduced accuracy on day 42 (c-statistic = 0.795). When compared to the discriminative model (c-statistic = 0.765) that only used birth weight as inputs, the metabolic-based model continuously revealed increased discriminative ability over the first six weeks of life.

## 4. Discussion

In this study we identified distinct longitudinal patterns of fatty acid and protein metabolism intermediates associated with infants who develop BPD among premature newborns. The derived metabolic panels revealed the longitudinal progressive metabolic deviation of BPD babies in the blood over the first six weeks of life after birth and thus potentially described early biologic clues of BPD during its development and newborn clinical management.

Prior studies have documented the importance of optimized nutrition on lung development in order to achieve normal lung structural morphology [21,22]. Reduced daily or cumulative calorie intake has been observed to be associated with increased odds of BPD in preterm infants for decades [23,24]. Postnatal nutritional deficits have also been recognized as independent predictors of BPD in extremely premature babies (i.e., GA ≤ 28 weeks) [25]. In addition, increased energy expenditure and elevated metabolic demands are often observed in preterm infants who develop BPD [26,27]. The interplay of increased energy expenditure and reduced caloric intake could result in short and long-term growth failures/deficits of infants who develop BPD [27,28]. However, GA and birth weight are important confounders and are the most important factors predicting postnatal growth faltering. As showed in our study, although reduced calorie intake and lower body weight were observed for BPD infants in the primary cohort, such postnatal deficits in infants who developed BPD were well adjusted and controlled after matching their GA with control infants in the sub-cohort. Therefore, considering the complex and multifactorial pathogenesis of BPD, more evidence is needed to explore whether caloric intake deficit and growth restriction are drivers of lung injury or more likely symptoms/complications during the development of BPD.

In this study, 27 distinct intermediates of fatty acid and protein metabolism were identified that were associated with the development of BPD over the early days of life, after adjusting for both GA and birth weight. At the first day of life, 0 metabolic differentials were captured. Subsequently, the number of identified AC and AA differentials progressively increased to 19 at day 28 of life, suggesting postnatal metabolic reprogramming for amino-acids, acylcarnitine, and fatty acids occurring progressively in response to clinical care and disease evolution [12].

The identified abnormal acylcarnitine profiles over the first months of life in our data imply a critical role of mitochondrial fatty acid β-oxidation in moderating lung injury and maintaining pulmonary function, where carnitine is a key molecule for infant energy utilization as it transports long chain fatty acids across the mitochondrial membrane [29]. In our study, levels of carnitine and most short- and median-chain ACs were reduced, whereas the levels of several long-chain acylcarnitines were increased in preterm newborns with BPD. This may reflect the dysregulated metabolism of fatty acids with different chain lengths in BPD. It is possible that the carnitine shuttle is impaired in BPD, making it unable to transport long-chain fatty acids into mitochondria for β-oxidation, leading to the accumulation of some long-chain acylcarnitines. In prior studies, acylcarnitine patterns in fibroblasts were found to be similar between patients with respiratory chain defects and those with fatty acid β-oxidation disorders [30]. Moreover, several studies have reported that a lack of carnitine contributes to the pathogenesis of respiratory disorders in preterm infants like respiratory distress syndrome, which may be further exacerbated by the excessive consumption of carnitine for surfactant synthesis in lung tissue [31]. Meanwhile, numerous prior studies have also revealed that the immaturity of lung macrophages and the involved metabolic reprogramming contributed to the development of BPD, since pro-inflammatory macrophages tend to utilize aerobic glycolysis to rapidly generate cytokines and employ mitochondrial respiration to fuel inflammatory responses [32,33,34,35]. In addition, the long-chain AC, C16_OH, was notably higher in BPD infants than controls in both our result and a previous BPD biomarker study [16]. We speculate that this may be explained in part by growing evidence that long-chain acyl-CoA dehydrogenase deficiency and a subsequent increase in long-chain ACs will inhibit the pulmonary surfactant and reduce lung function [36,37]. However, compared with the control group, not all of the long-chain ACs in BPD infants showed an increase in our data, thus additional studies are needed to more precisely identify the accompanying metabolic derangement.

Regarding protein metabolism, it has been previously shown that citrulline and tyrosine levels were noted to be significantly lower in infants with pulmonary hypertension secondary to BPD, while phenylalanine was increased in infants with persistent pulmonary hypertension [13,17,38]. In this study, these prior findings were replicated. We also found slightly reduced arginine in infants who developed BPD since day 28 of life, similar to the findings of other studies that reduced arginine and citrulline were associated with pulmonary hypertension and BPD [14]. Urea cycle metabolism involving citrulline is considered a primary biologic link between lung function and metabolism, as the cycle supplies the strong pulmonary vasodilator and alveolar growth promoter, nitric oxide (NO). NO is synthesized in pulmonary vascular endothelial cells from the coversion of L-arginine to NO and L-citrulline by NO synthase (NOS) [39,40]. Under pathophysiologic conditions, the excess induction of arginase isozymes could lead to unbalanced competition for the substrate, limiting the available L-arginine for NO synthesis [41]. Moreover, the genetic deficiency or decreased activity of urea cycle enzymes (like carbamoyl-phosphate synthetase, CPS) could also lead to insufficient substrate and downstream intermediates in the urea cycle, including citrulline. Therefore, as previously suggested, L-citrulline or L-arginine supplementation may be an effective therapy for the prevention of BPD [42,43]. Prior studies have further suggested that decreased levels of tyrosine may signal increased oxidative stress in infants developing BPD; accordingly antioxidants can affect newborns’ lung growth and development [17,44]. This can occur as a result of oxidative stress, the overproduction of superoxide leading to higher levels of peroxynitrite, which further reacts with tyrosine to produce 3-nitrotyrosine [45]. Thus, a high level of 3-nitrotyrosine but low levels of tyrosine has been recognized as an indicator of oxidative distress and the development of BPD [46]. In addition, increased phenylalanine level in BPD infants can also be interpreted as a result of tetrahydrobiopterin (BH4) depletion caused by oxidative stress [17,47,48]. A lack of BH4 would reduce the activity of phenylalanine hydroxylase (PAH), which converts phenylalanine to tyrosine, leading to both an increases in phenylalanine and a decrease in tyrosine [11,49].

A limitation of this study is that metabolic samples and clinical variables were exclusively collected at four time points over the first six weeks of life; thus it is possible that an element of time-dependent insight and subtle metabolic and clinical alterations may be attenuated or masked in this study. Second, in addition to the 72 targeted metabolic intermediates based upon a standard newborn screen at the time of collection in this study, additional metabolites and nutritional parameters should be captured in future studies to more deeply evaluate metabolic pathways discussed in this study, such as the urea cycle and fatty acid β-oxidation for an evaluation of their potential critical roles in the BPD pathogenesis. Thirdly, in this study, these metabolites are measured in the blood, while it is still unclear how the changes of these metabolites in the blood could reflect their levels in the lung. Fourthly, in this study, the metabolic indicators associated with BPD development were determined from infants with a lower GA (i.e., 27–28 weeks); thus our findings may only be applicable to extremely premature babies.

In this study, we identified a panel of fatty acid and protein metabolites associated with newborns who develop BPD. This panel described distinct longitudinal metabolic differences between infants with and without BPD in the blood from birth to day of life 42, independent from GA and birth weight. Several underlying metabolic pathways were discussed, implying their potential association with BPD development for extremely premature infants. However, it is still unclear whether there are similar changes of these metabolites in tracheal aspirates from infants with BPD. Therefore, we hope that our results could provide clues for early-life metabolic status in the blood during BPD pathogenesis and promote metabolic monitoring or investigation in the lung and other relevant tissues to benefit postnatal nutritional administration for future BPD prevention or management.

## Figures and Tables

**Table 1:** Demographics and characteristics of the sub-cohort with GA of 27–28 weeks.

Characteristics	Control (*n* = 108)	BPD (*n* = 97)	*p*-Value (BPD vs. Control)
Sex, *n* (%)			0.599
Male	44 (40.74%)	44 (45.36%)	
Race, *n* (%)			0.100
American Indian	4 (3.07%)	2 (2.06%)	
Asian	4 (3.07%)	4 (4.12%)	
Black	28 (25.93%)	19 (19.59%)	
Hispanic	15 (13.89%)	5 (5.15%)	
Other	0 (0.00%)	1 (1.03%)	
Pacific Islander	1 (0.93%)	0 (0.00%)	
White	56 (51.85%)	66 (68.04%)	
Mode of delivery, *n* (%)			0.944
Cesarean section	82 (75.93%)	75 (77.32%)	
Vaginal	26 (24.07%)	22 (22.68%)	
Multiple gestation, *n* (%)			0.790
One	85 (78.70%)	77 (79.38%)	
Two	22 (20.37%)	18 (18.56%)	
Three	1 (0.93%)	2 (2.06%)	
Antenatal steroids, *n* (%)			0.890
None	13 (12.04%)	9 (9.28%)	
Betamethasone	86 (79.63%)	80 (82.47%)	
Dexamethasone	7 (6.48%)	7 (7.22%)	
Both	2 (1.85%)	1 (1.03%)	
Birth weight (g), mean (SD)	1071 (210)	968 (198)	<0.001
Patent ductus arteriosus (PDA), *n* (%)	34 (31.5%)	53 (54.6%)	0.0011
Small for gestational age (SGA), *n* (%)	10 (9.26%)	10 (10.31%)	0.800
Apgar at 1 min, median (25%-75%)	6 (3–7)	5 (3–6)	0.113
Apgar at 5 min, median (25%-75%)	8 (7–8)	7 (6–8)	0.063

## Data Availability

The data presented in this study are available on request from the corresponding author.

## Supplementary Materials

The following supporting information can be downloaded at: https://www.mdpi.com/article/10.3390/nu14173547/s1. Figure S1: Study design. 351 data points of 97 BPD cases and 407 data points of 108 controls were collected longitudinally to identify metabolic markers. The gestational ages of these recruited infants ranged from 27 to 28 weeks; Figure S2: The p-value plot of the initial 72 metabolic variables, representing their difference between infants that developed BPD and controls at each time point. The dash line (1.96) is the significant level when using global FDR (0.05) correction for p-value; Figure S3: The calculated pairwise correlation of each two of the significant metabolites over the 4-time points in each of the two subgroups of BPD cases and controls; Table S1: Demographics and characteristics of the primary study cohort; Table S2: The list of 27 significant metabolic variables that were associated with BPD development at at least one time point, adjusted by birth weight and screened by the global FDR of 0.05, using the 27-28-week GA sub-cohort; Table S3: The list of 25 distinct metabolites that were extracted from the metabolic panel of the identified 27 metabolic variables, along with their class and annotations.

## Abbreviations

BPD	bronchopulmonary dysplasia (chronic lung disease of prematurity)
AA	amino acid
AC	acylcarnitine
NEC	necrotizing enterocolitis
IVH	intraventricular hemorrhage
ROP	retinopathy of prematurity
GA	gestational age
