# Supplementary material for: Progressive Metabolic Abnormalities Associated with the Development of Neonatal Bronchopulmonary Dysplasia
Source: Nutrients. 2022 Aug 28;14(17):3547. doi: 10.3390/nu14173547 (PMC9459725; doi:10.3390/nu14173547)
Supplement: Supplementary file 1 [file nutrients-14-03547-s001.zip › nutrients-1871823-supplementary.pdf]

Supplementary Figures and Tables

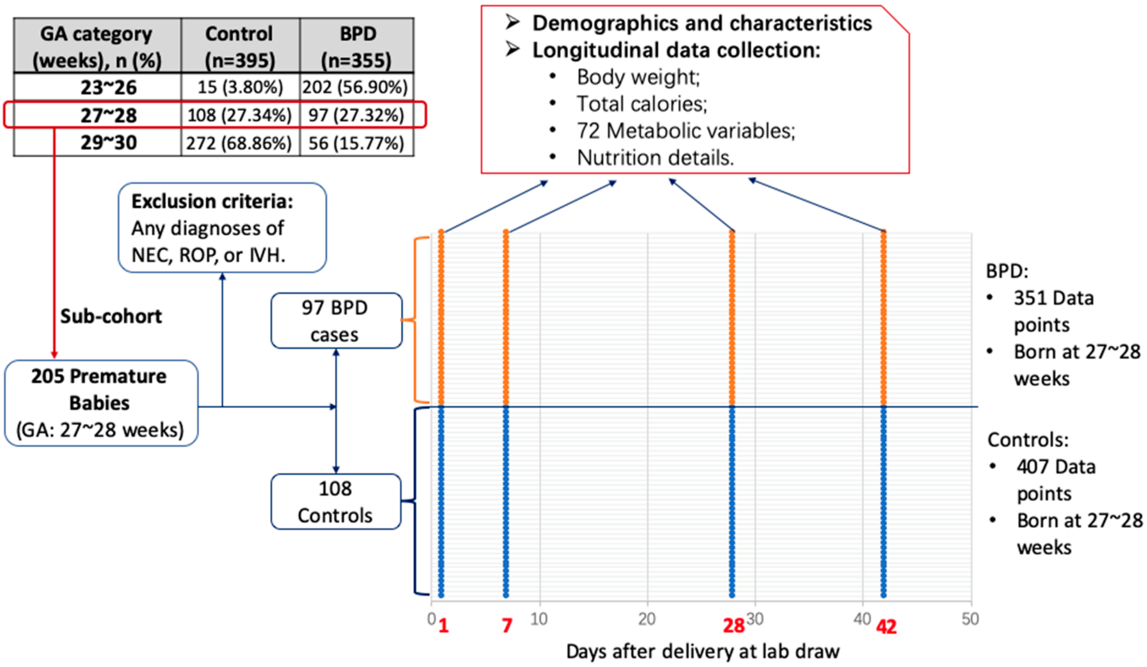

Figure S1. Study design. 351 data points of 97 BPD cases and 407 data points of 108 controls were collected longitudinally to identify metabolic markers. The gestational ages of these recruited infants ranged from 27 to 28 weeks.

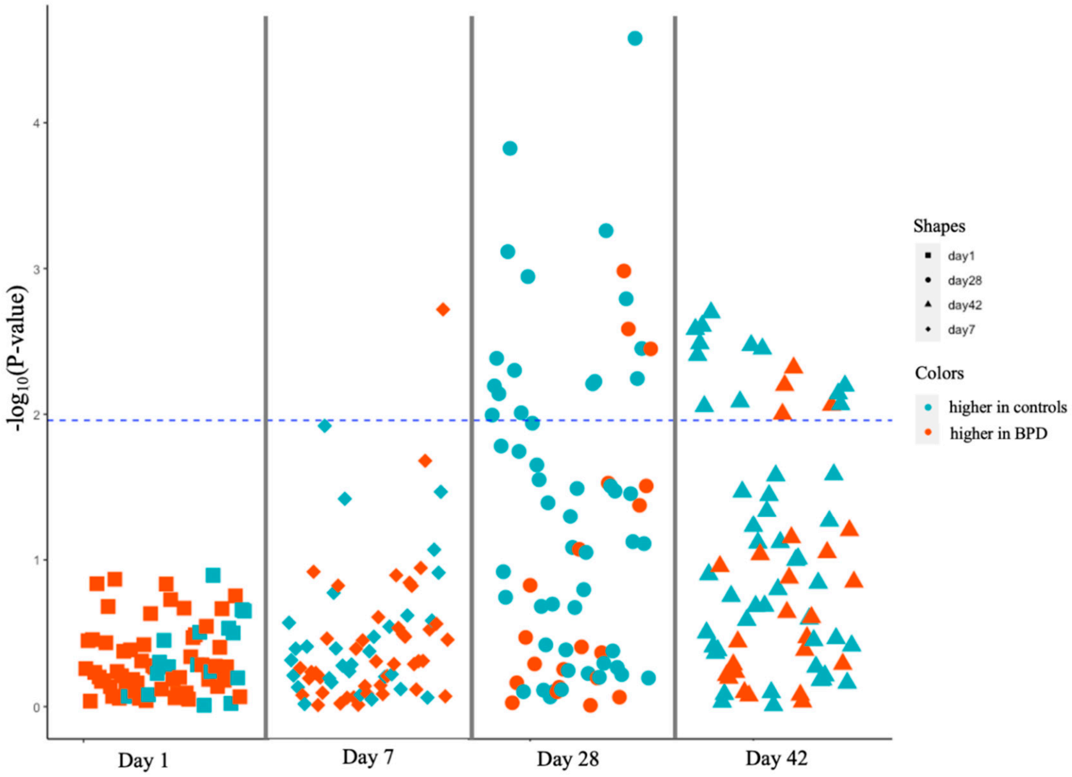

Figure S2. The p-value plot of the initial 72 metabolic variables, representing their difference between infants that developed BPD and controls at each time point. The dash line (1.96) is the significant level when using global FDR (0.05) correction for p-value.

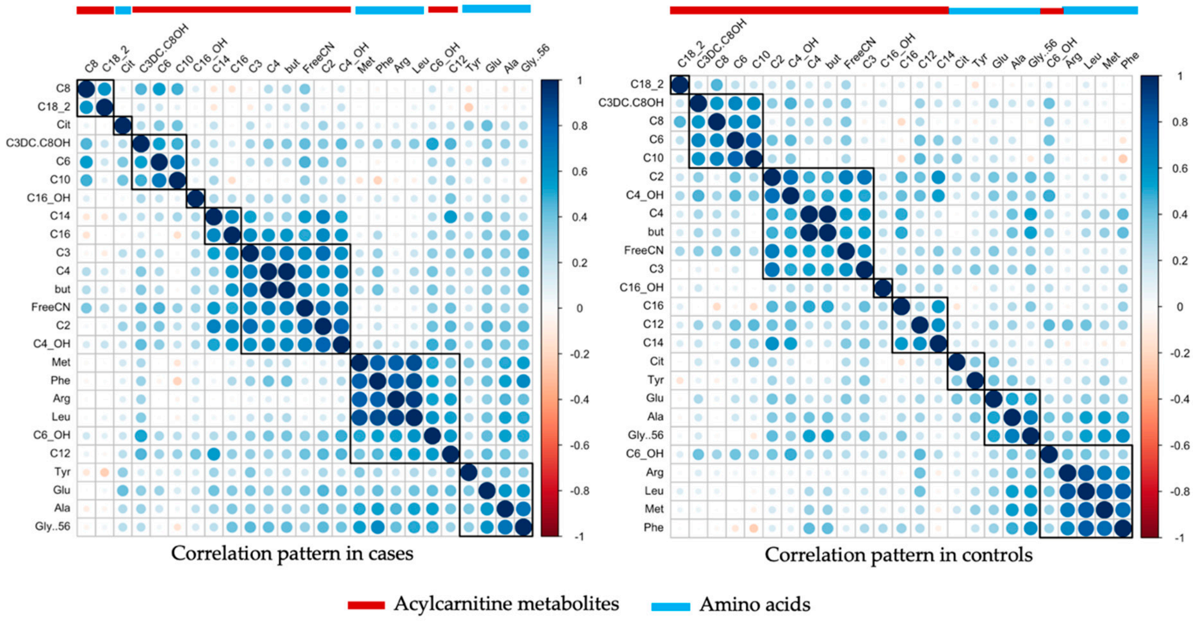

Figure S3. The calculated pairwise correlation of each two of the significant metabolites over the 4-time points in each of the two subgroups of BPD cases and controls.

Table S1. Demographics and characteristics of the primary study cohort.

Characteristics	Control (n=395)	BPD (n=355)	P-value (BPD vs. Control)
Sex, n (%)			0.926
Male	196 (49.62%)	174 (49.01%)	
Race, n (%)			0.9
American Indian	8 (2.03%)	4 (1.13%)	
Asian	12 (3.04%)	10 (2.82%)	
Black	96 (24.30%)	88 (24.79%)	
Hispanic	37 (9.37%)	33 (9.30%)	
Other	3 (0.76%)	5 (1.41%)	
Pacific Islander	1 (0.25%)	2 (0.56%)	
White	238 (60.25%)	213 (60.00%)	
Mode of delivery, n (%)			0.484
Cesarean section	297 (75.19%)	258 (72.68%)	
Vaginal	98 (24.81%)	97 (27.32%)	
Multiple gestation, n (%)			0.4
One	276 (69.87%)	264 (74.37%)	
Two	106 (26.84%)	81 (22.82%)	
Three	13 (3.29%)	10 (2.82%)	
GA category (weeks), n (%)			<0.001
23~26	15 (3.80%)	202 (56.90%)	
27~28	108 (27.34%)	97 (27.32%)	
29~30	272 (68.86%)	56 (15.77%)	

Birth weight (g), mean (SD)	1250.59 (289.11)	881.46 (262.18)	<0.001
Patent ductus arteriosus (PDA), n (%)	92 (23.3%)	227 (63.9%)	<0.001
Small for gestational age (SGA), n (%)	36 (9.11%)	34 (9.58%)	0.828
Apgar at 1 minute, median (25%-75%)	6 (4-7)	5 (2-6)	<0.001
Apgar at 5 minutes, median (25%-75%)	8 (7-9)	7 (6-8)	<0.001

Table S2. The list of 27 significant metabolic variables that were associated with BPD development at at least one time point, adjusted by birth weight and screened by the global FDR of 0.05, using the 27-28-week GA sub-cohort.

**Significant metabolic variables (n=27) after birth weight adjustment**		**Day**			
		**1**	**7**	**28**	**42**
**C2**	Acetylcarnitine			√	√
**C2_Glu**	Acylcarnitine + glutamate			√	√
**C3**	Propionylcarnitine			√	√
**C3_Met**	Propionylcarnitine + methionine			√	√
**C3DC.C8OH**	Malonylcarnitine (C3-DC) + 3-hydroxyoctanolylcarnitine (C8-OH)			√	
**C4**	Butyrylcarnitine + isobutyrylcarnitine				√
**C4_C3**	Butyrylcarnitine + isobutyrylcarnitine/propionylcarnitine ratio				√
**C4_OH**	Hydroxybutyrylcarnitine			√	√
**C6**	Hexanoylcarnitine			√	
**C6_OH**	Hydroxyhexanoylcarnitine			√	
**C8_C10..PR**	Octanoylcarnitine/decanoylcarnitine ratio		√		
**C8_C3**	Octanoylcarnitine/propionylcarnitine ratio				√
**C10**	Decanoylcarnitine			√	
**C12**	Dodecanoylcarnitine				√
**C14**	Tetradecanoylcarnitine				√
**C16_OH_C16**	Hydroxypalmitoylcarnitine/palmitoylcarnitine ratio				√
**C18_2**	Linoleoylcarnitine				√
**FreeCN**	Free carnitine			√	√
**FreeCN_C16**	Free carnitine/palmitoylcarnitine ratio			√	
**Ala**	Alanine			√	
**Cit**	Citrulline			√	
**Cit_Phe..119**	Citrulline/phenylalanine ratio			√	√
**Gly..56_Ala**	Gylcine/alanine ratio			√	
**Leu_Ala**	Leucine-isoleucine/alanine ratio			√	√
**Leu_Phe**	Leucine-isoleucine/phenylalanine ratio			√	
**Phe_Tyr**	Phenylalanine/tyrosine ratio			√	
**Cit.119._Arg**	Citrulline / arginine ratio			√	√

Total	0	1	19	16

Table S3. The list of 25 distinct metabolites that were extracted from the metabolic panel of the identified 27 metabolic variables, along with their class and annotations.

Reduced metabolite list (n=25)		Class	Annotations
**Phe**	Phenylalanine	**AA_1**	Glucogenic and ketogenic AA
**Tyr**	Tyrosine	**AA_1**	Glucogenic and ketogenic AA
**Leu**	Leucine	**AA_2**	Ketogenic AA
**Arg**	Arginine	**AA_3**	Glucogenic AA
**Ala**	Alanine	**AA_3**	Glucogenic AA
**Glu**	Glutamate	**AA_3**	Glucogenic AA
**Gly..56**	Glycine	**AA_3**	Glucogenic AA
**Cit**	Citrulline	**AA_3**	Glucogenic AA
**Met**	methionine	**AA_3**	Glucogenic AA
**FreeCN**	Free carnitine	**AC_1**	Carbons: 7-12
**C2**	Acetylcarnitine	**AC_1**	Carbons: 7-12
**C3**	Propionylcarnitine	**AC_1**	Carbons: 7-12
**But**	Butyrylcarnitine	**AC_1**	Carbons: 7-12
**C4**	Butyrylcarnitine + isobutyrylcarnitine	**AC_1**	Carbons: 7-12
**C4_OH**	Hydroxybutyrylcarnitine	**AC_1**	Carbons: 7-12
**C6**	Hexanoylcarnitine	**AC_2**	Carbons: 13-18
**C6_OH**	Hydroxyhexanoylcarnitine	**AC_2**	Carbons: 13-18
**C8**	Octanoylcarnitine	**AC_2**	Carbons: 13-18
**C10**	Decanoylcarnitine	**AC_2**	Carbons: 13-18
**C3DC.C8 OH**	Malonylcarnitine (C3-DC) + 3-hydroxyoctanolylcarnitine (C8-OH)	**AC_2**	Carbons: 13-18
**C12**	Dodecanoylcarnitine	**AC_3**	Carbons: 19-25
**C14**	Tetradecanoylcarnitine	**AC_3**	Carbons: 19-25
**C16_OH**	Hydroxypalmitoylcarnitine	**AC_3**	Carbons: 19-25
**C16**	Palmitoylcarnitine	**AC_3**	Carbons: 19-25
**C18_2**	Linoleoylcarnitine	**AC_3**	Carbons: 19-25
